# A 5-year course of predominantly obsessive vs. mixed subtypes of obsessive-compulsive disorder

**DOI:** 10.4103/0019-5545.37664

**Published:** 2007

**Authors:** S. B. Math, Jaideep Thoduguli, Y. C. Janardhan Reddy, P. N. Manoj, A. Zutshi, R. P. Rajkumar, A. M. Adarsh

**Affiliations:** OCD Clinic, Department of Psychiatry, National Institute of Mental Health and Neurosciences (NIMHANS), Bangalore - 560 029, Karnataka, India

**Keywords:** Course, obsessive-compulsive disorder, obsessive-compulsive disorder subtypes, outcome

## Abstract

**Background:**

Obsessive-compulsive disorder (OCD) is considered a heterogeneous disorder. One of the traditional approaches to subtype OCD is based on the predominance of obsessions, compulsions or both. Some studies suggest that the “predominantly obsessive” subtype of OCD may have poor outcome, whereas few other studies suggest that “mixed” OCD is associated with poor outcome. Therefore, it is not clear if the long-term course of “predominantly obsessive” subjects is different from those with “mixed” OCD. In the establishment of diagnostic validity of psychiatric conditions, differential course is an important validating factor.

**Aim:**

This study compares the 5-6 year course of the “predominantly obsessive” subtype with that of the “mixed” subtype of OCD with the objective of determining if the course of OCD differs according to subtypes and whether course could be a validating factor for subtyping OCD based on predominance of obsessions, compulsions or both.

**Setting and Design:**

Tertiary hospital, institutional setting. The study has a retrospective cohort design.

**Materials and Methods:**

Fifty-four subjects with “predominantly obsessions” and an equal number of the “mixed” subtype of OCD were recruited from the database of a specialty OCD clinic of a major psychiatric hospital. They were followed up after 5-6 years. The Yale-Brown Obsessive-Compulsive Scale (Y-BOCS) checklist and severity rating scale was used for assessing OCD. The course of OCD was determined according to predefined criteria.

**Statistics:**

The Chi-square/Fisher's exact test and the independent samples “*t*” test were used to compare categorical and continuous variables, respectively. Correlations were tested using the Pearson's correlation analysis.

**Results:**

Thirty-eight “predominantly obsessive” (70%) and 39 “mixed” (72%) OCD subjects could be traced and evaluated. The course of illness was similar in the two subtypes. A majority of the sample (72%) did not have clinical OCD at follow-up.

**Conclusions:**

“Predominantly obsessive” subjects have a course similar to those with “mixed” OCD. Clinically, it is reassuring to know that obsessive subjects do not have an unfavorable course as was suggested by some previous studies. In this sample, course did not validate the subtyping method employed, but it would be premature to conclude that the subtyping method employed is incorrect based on the course alone. Prospective study of the course in larger samples and neurobiological and family-genetic data may help further validation.

Obsessive-compulsive disorder (OCD) is considered a heterogeneous disorder, and there have been several attempts to subtype the disorder based on phenomenology, comorbidity, age-at-onset and family history.[1,2] Subtyping of OCD is important because homogenous samples may help in the elucidation of etiopathogenesis and development of newer and more effective treatment strategies. One such attempt to subtype OCD is based on the predominance of obsessions, compulsions or both. The International Classification of Diseases on Mental Disorders, 10^th^ revision (ICD-10) has adopted such an approach.[3] However, the findings of the DSM-IV field trial on ICD-10 subcategories of OCD were equivocal.[4]

Recently, there is some evidence from factor and cluster analytical studies that “predominantly obsessives” constitute a valid subgroup of OCD subjects.[5–9] Some studies suggest that they respond poorly to treatment, behavior therapy in particular, implying poor outcome.[10–15] On the contrary, few other studies have demonstrated that “mixed” OCD is associated with treatment nonresponse[16–18] and poor outcome.[19,20] Overall, there is limited data on the “predominantly obsessive” subjects. It is not known if the long-term course of “predominantly obsessive” subjects is different from those with “mixed” OCD. Our study compares the 5-6 year course of “predominantly obsessive” subjects with that of “mixed” OCD subjects in a retrospective cohort design. The objective is to examine if the course of OCD differs according to the predominance of obsessions, compulsions or both and whether course validates the subtyping method employed. However, we do not have “predominantly compulsive” subjects in this study since no subject received such a diagnosis in our clinical setting.

## MATERIALS AND METHODS

We conducted the study according to the guidelines of the Ethics Committee of the institute. Assessments were performed after obtaining written informed consent.

### Subjects

The study included 108 DSM-IV adult OCD subjects recruited from a total sample of 346 OCD subjects who attended the specialty OCD clinic of a major psychiatric hospital in the years 1999 and 2000.[21] We included *all* the “predominantly obsessive” subjects (*n* = 54) and for comparison, we randomly selected 54 subjects from the remaining sample of 292 “mixed” OCD subjects. There was none with the “predominantly compulsive” subtype of OCD. Not all the “mixed” subjects could be included because of the potential logistic problems in contacting and assessing such a large sample. The “mixed” OCD subjects included in the study (*n* = 54) did not differ significantly from those excluded (*n* = 238) with respect to age, age-at-onset of OCD, duration of illness, Yale-Brown Obsessive-Compulsive Scale (Y-BOCS)[22,23] severity scores, gender, marital and domicile status (urban/rural) and number and type of comorbid conditions.

Subtyping of OCD was based on the ICD-10 subcategories.[3] We also added a criterion: to be classified as “predominantly obsessive”, the Y-BOCS obsessions subscore should be >8 and compulsions subscore not greater than 5; to be classified as “predominantly compulsive”, the Y-BOCS compulsions subscore should be >8 and obsessions subscore not greater than 5; and “mixed” OCD subjects should have both obsessions and compulsions and score >15 on the Y-BOCS severity scale. We used a cutoff subscore of 5 for subtyping to ensure that the compulsions (in predominantly obsessives) and obsessions (in predominantly compulsive subjects) when present are not chief symptoms (in that they are not time-consuming and do not cause significant interference or distress). We chose this cutoff based on our clinical experience and to an extent, this subtyping method remains arbitrary. The criterion was added because the ICD-10 subcategories of OCD are not operationalized and therefore subtyping could become highly subjective and open to bias. The same definitions of OCD subtypes have been employed in a previous study.[16] Although mental compulsions are not recognized as compulsions in ICD-10, we have considered mental compulsions as equivalent of motor compulsions as in DSM-IV and subjects with obsessions and mental compulsions are classified as “mixed”.

The follow-up assessments were performed 5-6 years later in 2005 and 2006. Of the 54 “predominantly obsessive” subjects, we could trace and assess 38 subjects (70%); and of the 54 “mixed” OCD subjects, 39 (72%) could be evaluated. The evaluated obsessive subjects (*n* = 38) did not differ from the unevaluated obsessive subjects (*n* = 16) with respect to age, age-at-onset, gender, duration of illness, duration of untreated illness, marital status, domiciliary status, presence or absence of any comorbidity, family history of OCD and other psychiatric disorders and number of adequate trials with serotonin reuptake inhibitors (SRIs). They differed slightly with respect to patterns of occupation (*P* = 0.043). Similarly, the evaluated “mixed” OCD subjects (*n* = 39) did not differ significantly from the unevaluated “mixed” subjects (*n* = 15) on these variables except with respect to their domiciliary status (*P* = 0.044).

### Assessment

The subjects were extensively evaluated at baseline using a proforma (specially developed to assess OCD subjects) and the Y-BOCS symptom checklist and severity scale by postgraduate junior residents in psychiatry. A senior consultant expert in assessing OCD subjects further confirmed these findings. The evaluation included demographic details, obsessive-compulsive symptom profile, comorbid conditions, treatment details and family history of OCD and other major psychiatric illnesses.

At follow-up, either a psychiatric social worker or a psychiatrist trained in assessing the OCD subjects performed detailed unstructured clinical evaluations and administered the Y-BOCS scale. To assess the course in a systematic manner, the interviews typically began with the recapitulation of the problems at the time of initial consultation and then the subjects were asked to describe their symptoms in 1-year time intervals until the date of interview. We also used anchor points, such as major life events, academic years in college, calendar years, job changes, chronological age, major festivals and family events, to obtain the interval history. Detailed information was also obtained about the treatment received in the interim period. We reviewed the hospital clinical charts and the records of treatment received elsewhere to obtain supplemental history of the course of illness. Two senior psychiatrists of the OCD clinic reviewed all the available information and determined the course of OCD consensually according to the predetermined criteria.

### Course

The course of OCD was determined according to the following definitions used in two previous studies,[19,24] which are modifications of those employed by Thomsen.[25]

#### Chronic OCD

Symptoms persisted for most part of the course causing significant distress and impairment in functioning, satisfied the DSM-IV criteria for OCD.

#### Subclinical OCD

Mild symptoms, no significant distress or impairment in functioning for most part of the course and failed to satisfy the DSM-IV criteria.

#### Episodic OCD

Clear evidence of remissions (asymptomatic or subclinical) and relapses (significant distress and impairment in functioning) during the course of illness.

#### No OCD

No obsessive-compulsive symptoms after recovery from the index episode.

### True remission

Defined as “no OCD” status at follow-up without being on any treatment, i.e., being asymptomatic without any ongoing treatment. Those who are fully recovered (no OCD) and not taking treatment anymore belongs to this category.

### Statistical analysis

Categorical variables were compared using the Chi-square/Fisher's exact test and the continuous variables were compared using the independent samples “*t*” test. We did not employ *post hoc* correction despite multiple comparisons given the exploratory nature of the study. Correlations were tested using Pearson's correlation analysis. The statistical analyses were performed using the SPSS Version 13. The significance was set at *P*<0.05 (two-tailed)

## RESULTS

Demographic and certain clinical characteristics are given in Table 1. The predominantly obsessive subjects were older and had a later age-at-onset of illness. The two groups were treated similarly with SRIs, but more number of subjects in the mixed group received behavior therapy. The type of obsessions and compulsions (lifetime) and comorbid profile (lifetime) is given in Table 2. Major depression (lifetime) was more common in the “predominantly” obsessive group, but only showed a trend toward significance.

**Table 1:** Demoeraohic and illness characteristics

	n (%)/Mean (SD)		χ^2^/t	*P*-value
			
	Obsessive subjects (n = 38)	Mixed OCD subjects (n = 39)		
Age, range in years	40.1 (12.9), 19-70	32.6 (8.5), 21-55	3.012	0.004
Gender				
Male	25 (66)	28 (72)	0.324	0.569
Female	13 (34)	11 (28)		
Marital status				
Single	10 (26)	21 (54)	6.065	0.014
Married	28 (74)	18 (46)		
Occupation				
Student	5 (13)	2 (5)	12.385	0.030
Housewife	10 (26)	6 (15)		
Employee	13 (34)	16 (41)		
Business	6 (16)	6 (15)		
Agriculture	4 (11)	1 (3)		
Unemployed	0	8 (21)		
Locality				
Rural	9 (24)	9 (23)	0.009	0.995
Urban	22 (58)	23 (59)		
Semiurban	7 (18)	7 (18)		
Referral pattern				
Self	35 (92)	35 (90)	0.320	0.852
General practitioner	2 (5)	2 (5)		
Psychiatrist	1 (3)	2 (5)		
Years of education	12.4 (4.3)	12.7 (3.5)	2.086	0.7
Age at onset of illness	26.7 (11.7)	19.3 (6.8)	3.356	0.001
Duration of illness (months)	79.6 (90.1)	77.6 (77.5)	0.103	0.919
Duration of untreated illness (months)	71.2 (86.9)	58.9 (66.9)	0.701	0.486
Y-BOCS score, baseline				
Obsessions	12.7 (2.7)	14.1 (3.4)	2.086	0.04
Compulsions	1.1 (1.9)	13.3 (3.3)	19.708	<0.001
Total	13.7 (3.4)	27.4 (6.2)	12.155	<0.001
Number of SRI trials	2.1 (1.4)	2.5 (1.4)	0.767	0.142
Number of adequate SRI trials	1.6 (1.1)	1.8 (1.4)	0.160	0.497
Total number of patients who received augmentation	19 (50)	24 (62)	0.587	0.443
Atypical antipsychotics	2 (5)	9 (23)	4.592	0.032
Clomipramine	0	4 (10)	-	0.116†
Clonazepam	12 (32)	14 (36)	0.054	0.816
Buspirone	1 (3)	3 (10)	-	0.616†
Others	6 (16)	1 (3)	-	0.050†
Number on treatment currently with drugs	14 (39)*	20 (51)	1.160	0.281
Treatment with behavior therapy	2 (5)	10 (26)	5.619	0.018
Family history				
OCD	3 (8)	7 (18)	1.722	0.189
Others	11 (29)	15 (39)	0.779	0.337

OCD = Obsessive-compulsive disorder; Y-BOCS = Yale-Brown Obsessive Compulsive Scale; SRI = serotonin reuptake inhibitors,

* The percentage is calculated for 36 subjects instead of 38 because two subjects were deceased (one committed suicide and the other died of physical illness);

† Fisher's exact test

**Table 2:** Symptom profile and comorbidity

	n(%)		χ^2^	*P*-value
			
	Obsessive subjects (n = 38/54)	Mixed OCD subjects (n = 39/54)		
Obsessions				
Contamination	10 (26)	29 (74)	17.773	<0.001
Aggressive	21 (55)	21 (54)	0.16	0.901
Sexual	18 (47)	8 (21)	6.207	0.013
Religious	11 (29)	14 (36)	0.424	0.515
Hoarding	0	5 (13)	5.210	0.022
Pathological doubt	13 (34)	29 (74)	12.513	<0.001
Need for symmetry	3 (8)	12 (31)	6.420	0.011
Miscellaneous	28 (74)	20 (51)	4.114	0.043
Compulsions				
Washing	2 (5)	30 (77)	40.694	<0.001
Checking	9 (24)	28 (72)	17.847	<0.001
Repeating rituals	1 (3)	21 (54)	24.737	<0.001
Collecting	0	1 (3)	-	1.000
Ordering	0	9 (23)	-	0.002
Miscellaneous				
Mental rituals	4 (11)	15 (39)	8.081	0.004
Counting	1 (3)	5 (13)	-	0.200
List making	0	1 (3)	-	1.000
Slowness	0	2 (5)	-	0.494
Need to confess	0	1 (3)	-	1.000
Need to touch	0	2 (5)	-	0.494
Superstitious	1 (3)	4 (10)	-	0.358
Reassurance	1 (3)	5 (13)	-	0.200
Any other cognitive	3 (8)	5 (13)	-	0.711
Any comorbidity	21 (55)	17 (44)	6.33	0.426
Any tic disorder	1 (3)	2 (5)	-	1.000
MOD	17 (45)	10 (26)	3.082	0.079
Dysthymia	1 (3)	3 (8)	-	0.615
Bipolar	0	2 (5)	-	0.494
Panic disorder	2 (5)	1 (3)	-	0.615
Agoraphobia	1 (3)	0	-	0.494
GAD	0	1 (3)	-	1.000
ADS	1 (3)	0	-	0.494
Somatoform disorder	0	1 (3)	-	1.000
Any personality disorder	4 (11)	3 (8)	-	0.711

MDD = Major depressive disorder, GAD = Generalized anxiety disorder, ADS = Alcohol dependence syndrome

There was no statistically significant difference between the groups with respect to the course of illness [Table 3]. At follow-up, compared to baseline, there was a significant fall in the total Y-BOCS score in both the “predominantly obsessive” (13.67 ± 3.35 *vs.* 4.86 ± 4.94, *t* = 8.93, *P*<0.001) and “mixed” (27.41 ± 6.15 *vs.* 11.15 ± 10.47, *t* = 8.514, *P*<0.001) groups. The percentage of reduction in the Y-BOCS score was similar in both the obsessive (64%) and mixed (59%) groups. A majority did not have clinical OCD. Diagnostic stability was high in the “predominantly obsessive” group; only one subject developed “mixed” OCD during the course. One subject in each group also developed comorbid psychosis during the course of illness.

**Table 3:** Course of obsessive-compulsive disorder

	n (%)/Mean (SD)		χ^2^/t	*P*-value
			
	Obsessive group (n = 36)*	Mixed group (n = 39)		
Course†				
No OCD	15 (42)	13 (33)	1.215	0.545
Subclinical	13 (36)	13 (33)		
Chronic	8 (22)	13 (33)		
Prue remission‡	12 (33)	8 (21)	1.573	0.210

* Two subjects were deceased: One committed suicide and the other died of physical illness reducing the number of subjects with follow-up assessments to 36;

† None had episodic OCD;

‡ All true remitters are part of “No OCD” group

Because the two groups differed with respect to age and age-at-onset, we examined the correlation between these two variables and the Y-BOCS severity scores (baseline and follow-up) in both the groups using Pearson's correlation analysis. There was no significant correlation. Similarly, we compared the course between the two groups after eliminating those who had also received behavior therapy; there was no difference in the outcome (χ^2^ = 0.514, df = 2, *P* = 0.773)

## DISCUSSION AND CONCLUSION

Our study suggests that the course of “predominantly obsessive” and “mixed” OCD subtypes is similar. There is limited data on the course of the OCD subtypes. In the two long-term follow-up studies, “mixed” OCD was associated with poor outcome indicating better prognosis for those with “predominant obsessions”.[19,20] From the treatment response studies, there is conflicting evidence on the relationship between OCD subtypes and treatment response. Poor treatment response has been associated with both “predominantly obsessive”[10–15] and “mixed”[16–18] subtypes. Two studies found no relationship between symptom subtypes and medication response.[26,27] Interestingly, in a recent study, patients with “unacceptable thoughts”, a group with high levels of mental compulsions responded well to behavior therapy.[28] However, with the definition employed in our study, this group of patients with prominent mental compulsions may well get subtyped as mixed OCD. Our data, though not suggesting a better course in “predominantly obsessive” subjects, are definitely not indicative of poor course and outcome. It should be mentioned here that, in two previous studies, one a longitudinal follow-up study[19] and the other a study of predictors of non-response to drugs,[16] course was better in “predominantly obsessive” subjects. It is quite possible that sample attrition could have tilted balance in favor of “predominantly obsessive” subjects. Those with “mixed” OCD who had not improved may have been overrepresented in the two previous studies.

Our study finding needs to be interpreted with the definition of subtyping used. Previous studies did not employ ICD-10 OCD subtypes. In our study, in addition to using ICD-10 subtypes, we have employed the severity score on the Y-BOCS to define subtypes.[16] The ICD-10 provides subcategorization but does not define them. A related issue is the conceptualization of what constitutes “predominantly obsessive” subtype. There has been a tendency to classify subjects having obsessions and prominent mental compulsions as “obsessive” subtype, i.e., those without “overt compulsions”.[5] Even the cognitive behavior therapy (CBT) models for OCD mainly address “obsessive” subjects with mental compulsions.[28,29] However, the DSM-IV defines that mental compulsions are the equivalent of motor compulsions. Accordingly, in our study, subjects with obsessions and prominent mental compulsions received a diagnosis of “mixed” OCD.

The study has important implications. Clinically, it is reassuring to know that predominantly obsessives, at least, do as well as mixed OCD subjects considering that the existing literature suggests that they respond less well to treatment, particularly to CBT.[10–15] Subtyping of OCD is being actively pursued because homogenous samples help in the elucidation of etiopathogenesis and development of treatment strategies.[1,2] There is some evidence, at least from factor and cluster analytical studies that “predominantly obsessives” constitute a valid subgroup of OCD subjects.[5–9] In the establishment of diagnostic validity of psychiatric disorders, differential course is an important validating factor.[30] Our finding, however, suggests that the subtyping recommended in ICD-10 (but not defined) may not have predictive validity. The only other study, which examined the validity of ICD-10 subtyping, is the DSM-IV field trial on OCD.[4] The study examined the symptom profile and concluded that the results on the ICD-10 subtypes were equivocal with no clear implications. The field trial used the Y-BOCS checklist to categorize subtypes and the results on the subcategorization were equivocal with and without inclusion of mental compulsions in the compulsions category. The field trial did not examine the course. It would be, however, premature to conclude that obsessive subjects do not constitute a valid subtype based only on the course of illness. For example, the “predominantly obsessive” subjects in this study had more obsessions of “sexual” and “miscellaneous” type and less contamination fears, pathological doubts and concerns with order and symmetry. They also had a significantly later age-at-onset of illness with a trend toward a higher rate of comorbid depression.

The other criteria to validate the diagnosis proposed by Robins and Guze are equally important.[30] Future studies should focus on studying the subtypes in larger samples with a focus on neurobiological and family genetic parameters.[31] The findings of this study should be interpreted in the background of certain limitations. The sample size was relatively small and not all subjects could be assessed. The study was of a retrospective cohort with a catch-up longitudinal design. Therefore, it is obvious that elucidation of the course of OCD is heavily dependent on the recall of the subjects. We did not perform inter-rater reliability exercises. This may have not influenced the findings of the study significantly because two senior consultants reviewed all the available data and the course was determined consensually using predetermined criteria. Lastly, although the subjects were clinically well characterized in the specialty OCD clinic, the comorbid profile of OCD subjects was based on clinical interviews and not on structured assessments.
